# Substrate reduction therapy with miglustat for type 1 Gaucher disease: A retrospective analysis from a single institution

**DOI:** 10.3109/03009734.2011.641609

**Published:** 2012-02-15

**Authors:** Maciej Machaczka, Robert Hast, Ingrid Dahlman, Richard Lerner, Monika Klimkowska, Martin Engvall, Hans Hägglund

**Affiliations:** ^1^Division of Hematology, Department of Medicine at Huddinge, Karolinska Institutet, and Hematology Center Karolinska, Karolinska University Hospital Huddinge, Stockholm, Sweden; ^2^Department of Medicine at Solna, Karolinska Institutet, Stockholm, Sweden; ^3^Department of Endocrinology, Karolinska University Hospital Huddinge, Stockholm, Sweden; ^4^Department of Clinical Pathology and Cytology, Karolinska University Hospital Huddinge, Stockholm, Sweden; ^5^Centre for Inherited Metabolic Diseases, Karolinska University Hospital Huddinge, Stockholm, Sweden

**Keywords:** Adverse events, Gaucher disease, miglustat, substrate reduction therapy

## Abstract

**Introduction.:**

Gaucher disease (GD) is an infrequent progressive multisystem lysosomal storage disorder caused by the deficient activity of the lysosomal enzyme, glucocerebrosidase. A retrospective, single-center analysis of the clinical experience concerning the use of miglustat (N-butyldeoxynojirimycin), an oral inhibitor of glucosylceramide synthase, in type 1 Gaucher disease (GD1) was conducted to evaluate the efficacy, adverse events (AE), and outcome of miglustat therapy.

**Patients and methods.:**

Six adult Caucasian patients with GD1 (two women and four men), aged 21–81 years (median age 59 years), were treated with miglustat between October 2005 and April 2011. All but one patient (83%) carried at least one allele with c.1226A>G (N370S) mutation in the *GBA1* gene.

**Results.:**

Weight loss, diarrhea, poor appetite, and tremor were frequently reported AE by the patients. All of them experienced at least 2 AE, and three patients (50%) experienced at least 4 AE. Only two out of six patients (33%) have used miglustat longer than 12 months, of which only one used it longer than 15 months.

**Conclusions.:**

The major obstacle to successful miglustat therapy in GD1 was the high proportion of patients discontinuing their treatment due to the AE and the worsened quality of life. Further efforts are needed to improve tolerability of miglustat and, in consequence, compliance of patients treated with this orphan drug.

## Introduction

Gaucher disease (GD) is an infrequent progressive multisystem lysosomal storage disorder (LSD) caused by the deficient activity of the lysosomal enzyme, glucocerebrosidase (GBA), arising from autosomal recessive mutations in the *GBA1* gene (1q21) (1). There are more than 300 known mutations that can cause GD, among which the c.1226A >G (N370S) and the c.1448T>C (L444P) mutations are the most prevalent. Decreased GBA activity results in the accumulation of glucosylceramide in cells of the monocyte-macrophage system throughout the body (2,3). The clinical presentation of GD is highly variable. Classically, three clinical types of GD are distinguished according to the absence (type 1) or presence (types 2 and 3) of neurological symptoms and the dynamics of developing clinical signs. Thrombocytopenia, anemia, hepatosplenomegaly, and bone manifestations are the most typical signs of type 1 (GD1), the most prevalent form of GD (3). GD can be found in all ethnic groups. In Sweden, the overall prevalence of GD is approximately 1:170,000 individuals (unpublished own data), and this figure is slightly lower than GD prevalence reported in other Western countries but 2.5 times higher than in other Nordic countries (2,3).

Until 2010, two treatment options were available in Sweden for patients with GD: enzyme replacement therapy (ERT) with macrophage-targeted recombinant glucocerebrosidase (Cerezyme^®^, Genzyme Corporation, Cambridge, MA, USA), and substrate reduction therapy (SRT) with miglustat (Zavesca^®^, Actelion Pharmaceuticals, Allschwil, Switzerland). ERT was introduced for treatment of GD in 1991, and it is the standard of care for GD patients requiring treatment (2). ERT quickly and effectively improves hematological and visceral manifestations of GD, although its action on skeletal GD manifestations is slower, often taking many years before achieving improvement (4–7). SRT with N-butyldeoxynojirimycin (miglustat), a small iminosugar molecule, reversibly inhibits glucosylceramide synthase, the ceramide-specific glucosyltransferase that catalyzes the first committed step in glycosphingolipid synthesis, and in this way reduces intracellular storage of glucosylceramide (8). Miglustat is commercially available for the treatment of mild to moderate GD1 in the EU since 2002. Recent data confirmed miglustat efficacy in the long-term maintenance therapy of GD1 (9).

The purpose of our work was to evaluate retrospectively the efficacy and adverse events (AE) of miglustat therapy in adults with GD1 treated in the clinical practice setting.

## Patients and methods

There are currently 35 patients diagnosed with GD1 in Sweden. Between 2002 and 2010, 12 adults with GD1 were followed at Karolinska University Hospital in Stockholm, Sweden. Of these, six (50%) patients were temporarily or permanently treated with miglustat and were included in this analysis.

In all studied patients, the diagnosis of GD was confirmed by a low activity of glucocerebrosidase in peripheral blood leukocytes and increased activity of plasma chitotriosidase at a reference laboratory according to standard practice. Further direct DNA sequencing performed at the Academic Medical Center in Amsterdam, the Netherlands, revealed mutations in the *GBA1* gene in all cases.

Patients received commercially available miglustat capsules of 100 mg (Zavesca^®^, Actelion Pharmaceuticals) orally at a dose of 100 mg three times a day (t.i.d.). Recommendations concerning the correct administration of miglustat and the implementation of a low-carbohydrate diet (especially during the first weeks of treatment) were provided to all patients.

The efficacy of miglustat therapy was evaluated based on clinical examination and a comparison of blood GD markers measured at baseline (before starting miglustat) and at follow-up. Analyzed variables included plasma chitotriosidase activity (control range: <40 nkat/L), plasma concentration of chemokine (C-C motif) ligand 18/pulmonary and activation-regulated (CCL18/PARC) (control range: <100 μg/L), whole blood hemoglobin concentration (Hb) (control range: 117–153 g/L), and whole blood platelet count (PLT) (control range: 165–387 × 10^9^/L). Moreover, the serum concentration of ferritin (control range: 10–150 μg/L) and a profile of plasma immunoglobulin determined by plasma electrophoresis and immunofixation were followed up. Assessment of the aforementioned variables was performed at baseline and at 2, 4, 8, 12, and 15 months on miglustat therapy. Patients’ body weight was documented at the onset of and during miglustat therapy. AE occurring while on miglustat were reported by patients to their physicians and documented in patient files. The files were reviewed for collection of relevant data. Patients provided their informed consent.

## Results

Six adult Caucasian patients with GD1 (two women, four men), aged 21–81 years (median age 59 years), were treated with miglustat between October 2005 and April 2011. None of these patients had a known Jewish ancestry. All but one (83%) patient carried at least one allele with c.1226A>G (N370S) mutation in the *GBA1* gene. One patient with a more severe phenotype exhibited the heterozygous mutations c.798C>G and c.1040T>G in the *GBA1* gene by direct DNA sequencing, which to the best of our knowledge were never previously reported in patients with GD (1). Three (50%) patients were splenectomized earlier in their life due to GD. Patient characteristics are presented in Table I.

**Table I.** Patient characteristics.

Pt	Sex/Age (y)^a^	*GBA1* gene mutations	Age at Dx (y)	SPC/Age (y)	Duration of ERT (y)	SMG	BD
1	F/21	c.798C>G/c.1040T >G (novel mutations)	3	Y/5	4	NA	Y, s
2	M/42	c.437C>T/c.1226A >G (S107L/N370S)	3	Y/12	13	NA	Y, s
3	F/56	c.1226A>G/c.1226A >G (N370S/N370S)	55	N	NA	Y	Y, rs
4	M/62	c.721G>A/c.1226A >G (G202R/N370S)	61	N	1	Y	Y, s
5	M/65	c.1226A>G/RecNci (N370S/RecNci)	51	N	NA	Y	Y, rs
6	M/81	c.1226A>G/c.1448T >C (N370S/L444P)	30	Y/32	NA	NA	Y, rs

^a^Age at start of miglustat therapy.

Pt = patient; Dx = diagnosis of Gaucher disease; SPC = splenectomy; ERT = enzyme replacement therapy; SMG = splenomegaly; BD = bone disease (rs: radiological signs only; s: symptomatic disease); F = female; M = male; Y = yes; N = no; NA = not applicable.

### Initiation of miglustat therapy

Three patients (pts) were previously treated with ERT but wished to switch their treatment plan to a more convenient oral maintenance therapy. Occupation was a decisive factor for two frequently traveling patients (pts 2 and 4) and one student (pt 1) when choosing between oral versus intravenous therapy. They all wished to decrease contacts with the health care system to a minimum.

The remaining three patients were therapy-naive and began miglustat therapy as their initial treatment of GD. One patient (pt 6) refused the proposed ERT and began miglustat as a more convenient oral alternative. Two patients (pts 3 and 5) started miglustat as their first-line treatment due to the worldwide supply shortage of Cerezyme^®^ during 2009–2010 which was a consequence of viral contamination (vesivirus 2117) of the production plant in June 2009 (10).

### Efficacy of miglustat therapy

During the first 4 months of miglustat therapy, no changes in the levels of Hb and PLT were noticed in three patients (pts 3, 4, and 6). Hb was reduced by 12% in two patients (pts 1 and 5). In patient 1, PLT decreased by 35% after 4 months on miglustat but still remained within the normal range. On the contrary, PLT in patient 5 increased by 90% after the first 4 months of miglustat therapy, but at the time he was experiencing a strong inflammatory activity due to lung infection with *Mycobacterium avium* and reactivation of *Aspergillus fumigatus* in a lung aspergilloma (he earlier underwent partial resection of the right lung due to aspergilloma). Of note, patient 6 suffered from multiple myeloma of the IgG-lambda type before starting miglustat and was diagnosed with myelodysplastic syndrome (MDS) RAEB-2 (refractory anemia with excess of blasts type 2) 3 months after the start of miglustat (11). Therefore, a reliable interpretation of changes in the blood panel during miglustat therapy with respect to GD is not possible in his case.

One patient (pt 3) showed permanently reduced chitotriosidase activity as early as 2 months after the start of miglustat and a reduction of CCL18 concentration after 8 months of SRT. On the other hand, patient 1 with a novel heterozygous *GBA1* mutation and a more severe GD1 phenotype showed doubling of chitotriosidase activity 4 months after miglustat initiation. An objective interpretation of chitotriosidase and CCL18 alterations on miglustat therapy in the remaining patients is not possible because of short follow-up or untimely analyses. Ferritin concentrations were either stable (pts 3 and 2) or rising (pts 1, 2, and 5) over the period of miglustat treatment. In all but one patient (pt 6) plasma immunoglobulin profile was unchanged by miglustat therapy in respect to polyclonal or monoclonal gammopathy. The effects of miglustat therapy on visceral and skeletal disease are not discussed herein due to the short follow-up period of the majority of patients with respect to these GD manifestations.

Changes in blood markers of GD and selected variables significant in GD observed during miglustat therapy are given in Table II.

**Table II.** Changes in values of selected variables observed during miglustat therapy.

			Values of analyzed variables during miglustat therapy					
Pt	Sex/Age (y)^a^	Variables	0	2 m	4 m	8 m	12 m	15 m
1	F/21	B-Hb B-PLT S-Ferrititn P-Chito P-CCL18 Ig profile	122 260 202 1189 428 Normal	ND	108 170 636 2392 ND Normal			
2	M/42	B-Hb B-PLT S-Ferritin P-Chito P-CCL18 Ig profile	140 116 1255 ND ND ND	ND	ND	127 95 1627 ND ND ^P^ IgG 19.3	117 107 ND ND ND ND	127 119 2269 ND ND ^P^ IgG 18.6
3	F/56	B-Hb B-PLT S-Ferritin P-Chito P-CCL18 Ig profile	122 68 833 1549 908 ^P^ IgM 3.2	119 66 866 446 939 ^P^ IgM 3.1	NA	125 102 932 292 323 ^P^ IgM 3.6	NA	133 104 858 323 497 ^P^ IgM 3.4
4	M/62	B-Hb B-PLT S-Ferritin P-Chito P-CCL18 Ig profile	133 145 ND 3039 628 ^M^ IgM–λ 1.5	ND	136 145 ND ND ND ^M^ IgM–λ 2.0			
5	M/65	B-Hb B-PLT S-Ferritin P-Chito P-CCL18 Ig profile	124 104 1239 ND‡ ND‡ ^P^ IgG 14.9	115 180 2389 ND ND ND	109 198 4028 986 1062 ^P^ IgG 15.2			
6	M/81	B-Hb B-PLT S-Ferritin P-Chito P-CCL18 Ig profile	93 20 4408 2168 ND ^M^ IgG–λ 33	84 19 4047 ND ND ^M^ IgG–λ 25	90 12 3260 ND ND ^M^ IgG–λ 24	91 6 ND ND ND ND		

^a^Age at start of miglustat therapy.

^b^The samples were improperly transported to the laboratory.

Pt = patient; m = months on miglustat therapy; B-Hb = whole blood hemoglobin concentration (control range: 117–153 g/L); B-PLT = whole blood platelet count (control range: 165–387 × 10^9^/L); S-Ferritin = serum ferritin concentration (control range: 10–150 μg/L); P-Chito = activity of plasma chitotriosidase (control range: <40 nkat/L); P-CCL18 = concentration of plasma chemokine (C-C motif) ligand 18/pulmonary and activation-regulated (control range: <100 μg/L); ND = not determined; NA = not applicable (analyses done at the other laboratory); Ig = immunoglobulin (g/L); ^P^ = polyclonal; ^M^ = monoclonal.

### Adverse events during miglustat therapy

All six patients treated with miglustat suffered from at least 2 AE, and three (50%) patients experienced at least 4 AE (Table III). Four (66%) patients reported diarrhea, and three (50%) patients had poor appetite. Tremor appeared in five (83%) patients, but a more severe form was observed only in one patient (pt 2). Peripheral neuropathy (PN) was diagnosed in two (33%) patients; however, multifactorial etiology was considered in one patient (pt 6) who had a known mild PN, vitamin B12 deficiency, and MGUS/myeloma before the start of miglustat therapy. All six (100%) patients experienced negative weight change; three of them (pts 1, 5, and 6) lost ≥10% of their baseline body weight, and the remaining three patients (pts 2, 3, and 4) lost <10% of their baseline body weight (Table III).

**Table III.** Adverse events and body weight changes observed on miglustat therapy.

Pt	Sex/Age (y)^a^	Duration of SRT (months)	Adverse events	Absolute weight loss on SRT	Maximal percentage body weight change as compared to baseline
1	F/21	4	Diarrhea, poor appetite, weight loss	6 kg	–11%
2	M/42	15	Diarrhea, weight loss, tremor (moderate), peripheral neuropathy	4 kg	–5%
3	F/56	20^b^	Weight loss, tremor (mild)	5 kg	–8%
4	M/62	3	Weight loss, tremor (mild)	3 kg	–4%
5	M/65	4	Diarrhea, poor appetite, weight loss, tremor (mild)	10 kg	–16%
6	M/81	8^c^	Diarrhea, poor appetite, weight loss^d^, tremor (mild), peripheral neuropathy^d^	12 kg	–18%

^a^Age at start of miglustat therapy.

^b^Still on miglustat therapy.

^c^The pt died on ERT + SRT therapy due to acute myeloid leukemia.

^d^Possible multifactorial etiology (see comment in text).

Pt = patient; SRT = substrate reduction therapy with miglustat; F = female; M = male.

### Duration of miglustat therapy

Only two out of six (33%) patients used miglustat for longer than 12 months (pts 2 and 3), of which only one (17%) patient was treated for more than 15 months (pt 3).

Three patients (pts 1, 4, and 5) discontinued miglustat after 4 months of treatment due to AE negatively impacting their quality of life (QoL). Additionally, one patient (pt 5) discontinued miglustat due to co-morbidities (*Aspergillus fumigatus* and *Mycobacterium avium* infections). In one patient (pt 6), miglustat did not influence the course of his GD after 3 months of therapy, and he agreed to ERT. Nevertheless, combination therapy ERT + SRT was continued in the hope of achieving a rapid reduction in the bone marrow Gaucher cell burden, but shortly afterwards transformation to acute myeloid leukemia (AML) occurred and the patient died.

Patient 2 continued miglustat therapy for 15 months despite AE, although the compliance was suboptimal, with the use of reduced miglustat doses of 100 mg per day or 100 mg twice a day (b.i.d.), upon which gradual worsening of GD status was noticeable. He finally discontinued miglustat after 15 months.

Patient 3 considered miglustat to be a convenient therapy, and she is still using it in a full, prescribed dose more than 20 months after the start of SRT. She closely followed dietary restrictions for several months as recommended; however, she had mentioned on several occasions that to follow a Mediterranean-like diet with restrictions of lactose and disaccharides is quite expensive for an average person in Sweden. This opinion was shared by three other patients. Five (83%) patients stated that the recommended miglustat administration schedule (t.i.d.) was inconvenient for them as a form of daily life-long therapy as had been intended originally.

## Discussion

The experience of miglustat therapy for GD1 in the clinical practice setting has seldom been studied (9). The present study is a single-center report based on the experience gained with miglustat treatment in 6 out of 12 adults with GD1 (50%) controlled at Karolinska University Hospital, which constitutes 17% of the entire Swedish GD1 population (6/35 pts). The small patient number in this retrospective analysis may be perceived as a limitation; however, it depends on the rarity of inherited orphan diseases. The latter makes it often impossible to collect large, homogeneous patient groups to be in the same clinical situation at a single center, even over a long period of time.

Recent data on the long-term miglustat maintenance therapy of GD1 support a positive impact of miglustat on both bone marrow and bone tissue (8,9,12,13). Our analysis of GD markers and blood variables important for GD has confirmed the efficacy of miglustat in one treatment-naive patient as well as the probable non-inferiority of miglustat in one patient switched from ERT. An objective assessment of miglustat efficacy in the remaining four patients was not possible due to the short follow-up and the co-morbidities in two cases.

A major obstacle to successful miglustat therapy in GD1 is the relatively high proportion of patients discontinuing their treatment due to the worsened QoL (9,14). During the first weeks or months of miglustat therapy, a patient’s QoL is often affected by gastrointestinal AE (e.g. diarrhea, nausea, poor appetite, flatulence, and abdominal cramps), weight loss, and tremor (14,15). Miglustat inhibits digestive disaccharidases in the small intestinal mucosa: sucrase and maltase strongly, and lactase more weakly (16). In consequence, gastrointestinal AE in miglustat-treated patients are attributed to the suboptimal hydrolysis of carbohydrates, subsequent osmotic diarrhea, and altered colonic fermentation (15,16). Of note, gastrointestinal AE tend to decrease in intensity and frequency over time on continued miglustat therapy. In order to minimize weight loss and gastrointestinal AE, the producer (Actelion Pharmaceuticals) recommends that the miglustat-treated patients should follow a low-disaccharide and low-lactose diet, especially during the first weeks of therapy. The etiology of neurological side-effects associated with miglustat treatment (e.g. tremor and PN) is less understood. Generally, the profile and the frequency of AE observed in our miglustat-treated patients with GD1 are in line with the results of previously reported studies (8,9,14,15). Although the patients received dietary recommendations, all of them developed gastrointestinal AE affecting QoL, and as a consequence a half of them (three of six pts) discontinued miglustat treatment.

In the later period of miglustat treatment, QoL can be affected by the persistent AE from the early phase of therapy, new AE (e.g. PN), progression of GD due to poor compliance or an unsatisfactory disease response to miglustat, the busy miglustat administration schedule (t.i.d.), or issues related to dietary restrictions aimed at diminishing gastrointestinal AE (8,9,14). Worsened QoL can jeopardize a patient’s compliance, leading to therapy discontinuation. It should be appreciated that the producer is making efforts to improve the compliance of miglustat-treated patients in Sweden by supporting dietary issues, e.g. offering miglustat patients dietary brochures and a phone contact with a nurse in case of any dietary questions. However, dietary restrictions do not seem to be the ultimate solution of QoL-related issues in miglustat patients since they by themselves also limit QoL (dietary schedule, economic aspects) and do not influence non-gastrointestinal AE or the busy miglustat administration schedule.

There is no doubt that miglustat is an important alternative in the therapy of GD. An interesting feature of miglustat suggested by previous reports, particularly important in neuronopathic LSDs, is its potential to reach the brain, where miglustat may have beneficial effects on the defective metabolism of glycosphingolipids (17–20). However, the precise mechanism by which miglustat might cross the blood−brain barrier has yet to be established.

This report highlights that miglustat is a difficult drug to administer in the clinical practice setting. The overall impression is that GD1 patients experienced the negative impact of miglustat on their QoL early in the course of their treatment, resulting in a feeling of disappointment and ultimately in the discontinuation of therapy. Nevertheless, some possible areas of improvement can be identified, including further support to miglustat patients with simple and cost-effective dietary recommendations, a slow-release capsule formula for ‘once-a-day administration’ instead of the current ‘t.i.d. capsule’, and a focus on the understanding of the pathomechanisms behind the neurological symptoms in GD1 as well as measurements useful in their control (21,22). Moreover, further studies are needed to answer the question whether combination therapy ERT + SRT, ideally dose reduced and schedule modified as compared to monotherapy with both treatments, may circumvent obstacles seen with miglustat therapy in GD1. Such a combination therapy may offer GD patients better disease control (by employing more than one mechanism of action against the accumulation of glucosylceramide in cells), can be cost-effective by using reduced doses of both ERT and miglustat, and can provide an acceptable QoL.
